# Biological function of d-tryptophan: a bibliometric analysis and review

**DOI:** 10.3389/fmicb.2024.1455540

**Published:** 2025-01-13

**Authors:** Fei Wang, Runyu Du, Yunxiao Shang

**Affiliations:** ^1^Department of Pediatrics, Shengjing Hospital of China Medical University, Shenyang, China; ^2^Department of Endocrinology, Shengjing Hospital of China Medical University, Shenyang, China

**Keywords:** d-tryptophan, bibliometric analysis, d-amino acids, biological function, literature review

## Abstract

**Background:**

d-Tryptophan is recognised for its unique physiological properties. In this study, we aimed to explore the dynamic trends and emerging topics in d-tryptophan research to offer fresh perspectives for future studies.

**Methods:**

Employing bibliometric analysis, we examined the literature on d-tryptophan indexed in the Web of Science Core Collection from January 1987 to December 2023. The “Bibliometrix” R package and CiteSpace were utilised for data processing.

**Results:**

Analyses of 865 publications revealed 2209 keywords, 4068 authors, 2094 institutions, and contributors from 302 regions. The USA was at the forefront of publications concerning d-tryptophan, but the European Journal of Pharmacology, Journal of Biological Chemistry, and Journal of Medicinal Chemistry were notable for their contributions, co-citations, and impact, respectively. This literature review reveals that since 1987, studies have developed from a focus on d-tryptophan metabolism to the exploration of its functions in organic and medicinal chemistry and food science. Recent findings highlight the potential of d-tryptophan as a non-nutritional sweetener and food preservative as well as its role in inhibiting the growth of bacterial biofilms. Additionally, its immunomodulatory properties are being investigated in relation to allergic diseases. Furthermore, d-tryptophan plays a role in the therapy of atherosclerosis, osteoporosis, tuberculosis, and cancer.

**Conclusion:**

The results of bibliometric analysis highlight that future research should focus on the biological functions of d-tryptophan as a food preservative and its use in immunomodulation and drug development, providing strong guidance for future research.

## Graphical abstract

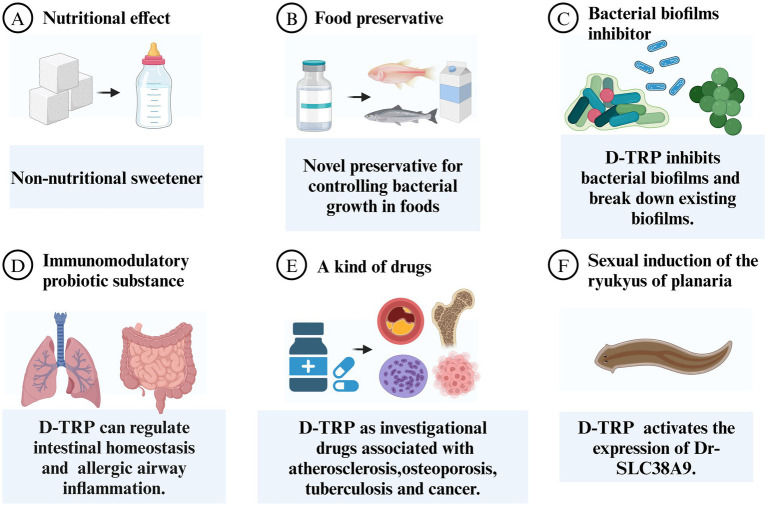

## 1 Introduction

d-Amino acids are enantiomers of l-amino acids and were thought to have no biological function in higher organisms until the late 1980s, when free d-aspartate and d-serine were discovered to act as neurotransmitters in mammalian brains (Hashimoto et al., 1993). d-Tryptophan, a non-protein, optically active amino acid, is an enantiomer of l-tryptophan and has unique physiological properties. Recent studies have shown that d-tryptophan acts as a bacterial metabolite (Kepert et al., 2017). d-Tryptophan is biosynthesized by microbes and can act as an immunomodulatory probiotic substance regulating intestinal homeostasis and helping to relieve allergic diseases, including allergic asthma (Kepert et al., 2017), and colonitis (Seki et al., 2022). In the pharmaceutical industry, d-tryptophan is an important synthetic precursor for drugs for cancer therapy (Hashemzadeh et al., 2021; Li et al., 2022; Liu H. et al., 2022; Liu Y. et al., 2022; Matheus et al., 2020; Zhang Y. et al., 2021), the treatment of atherosclerosis (Biros et al., 2022b), osteoporosis (Biros et al., 2022a), and tuberculosis (Singh et al., 2023).

Bibliometrics is a method of analyzing literature in a specific research field by assessing the quantity and quality of publications (Chen, 2004). This analysis provides a comprehensive understanding of authors, keywords, journals, countries, institutions, and references related to the research area (Chen, 2004). Bibliometric analysis tools such as CiteSpace (Sabé et al., 2023) and Bibliometrix (Li et al., 2020) in the R package allow the visualization of the results of bibliographic analysis.

Since 1987, research on d-tryptophan has gradually increased; however, there is a literature gap concerning dosimetric analysis and reviews of d-tryptophan. The purpose of this study was to conduct a quantitative analysis of research on d-tryptophan and to review current knowledge on d-tryptophan. This review systematically illustrates the research history and value of d-tryptophan in the food, feed, and pharmaceutical industries.

## 2 Bibliometric analysis

### 2.1 Search strategy

Data were acquired from the Web of Science Core Collection (1985–present) (WoSCC) database for this bibliometric analysis. The search methodology involved combining the following keywords and terms: “d-tryptophan OR d-Tryptophan OR d-Trp OR d-TRP OR d-trp.” The publication language was restricted to English and the time span was January 1987 to December 2023.

### 2.2 Study selection

All relevant publications were evaluated and collected by reading the titles and abstracts of WoSCC. Articles were included according to the following criteria: (1) the study involved d-tryptophan; (2) the main topic of the article was the application of d-tryptophan to a certain disease; (3) the study involved derivatives of D-tryptophan. Exclusion criteria: (1) the publications were letters, editorial materials, books, or unspecified studies. (2) the research areas did not pertain to the biological functions of d-tryptophan, such as technology, instrumentation, spectroscopy, engineering, and materials science.

### 2.3 Bibliometric data source

Bibliometric data were obtained from the WoSCC database from 1 January 1987 to 31 December 2023. Because obtaining high-quality and accurate analysis results is important, the publication language was restricted to English. All relevant documents were exported as “full records and references” in TXT format. The literature was first imported into Citespace for processing to remove duplicates.

### 2.4 Data analysis

After extraction, data analysis was performed using the “Bibliometrix” (Aria and Cuccurullo, 2017) package in R and CiteSpace (version 6.2. R6) (Chen and Song, 2019). The articles and author information and cooperation among countries were analyzed using “Bibliometrix.” CiteSpace was utilized to visualize the trends in keywords, and we mainly focused on identifying the time, frequencies, and centralities of keyword co-occurrence and category and keyword burst figures. We conducted keyword k-mean cluster analysis using a G-index value of k = 25 using Pathfinder to prune sliced and merged networks. CiteSpace was also used to visualize the dual-map overlay. Different clusters are represented by different colors. The size of the circles is positively correlated with the appearance frequency of the terms, and the thickness of the line indicates the strength of the connection between the terms.

### 2.5 Results

#### 2.5.1 Annual growth trends in publications and distribution of countries

**Figure 1:**
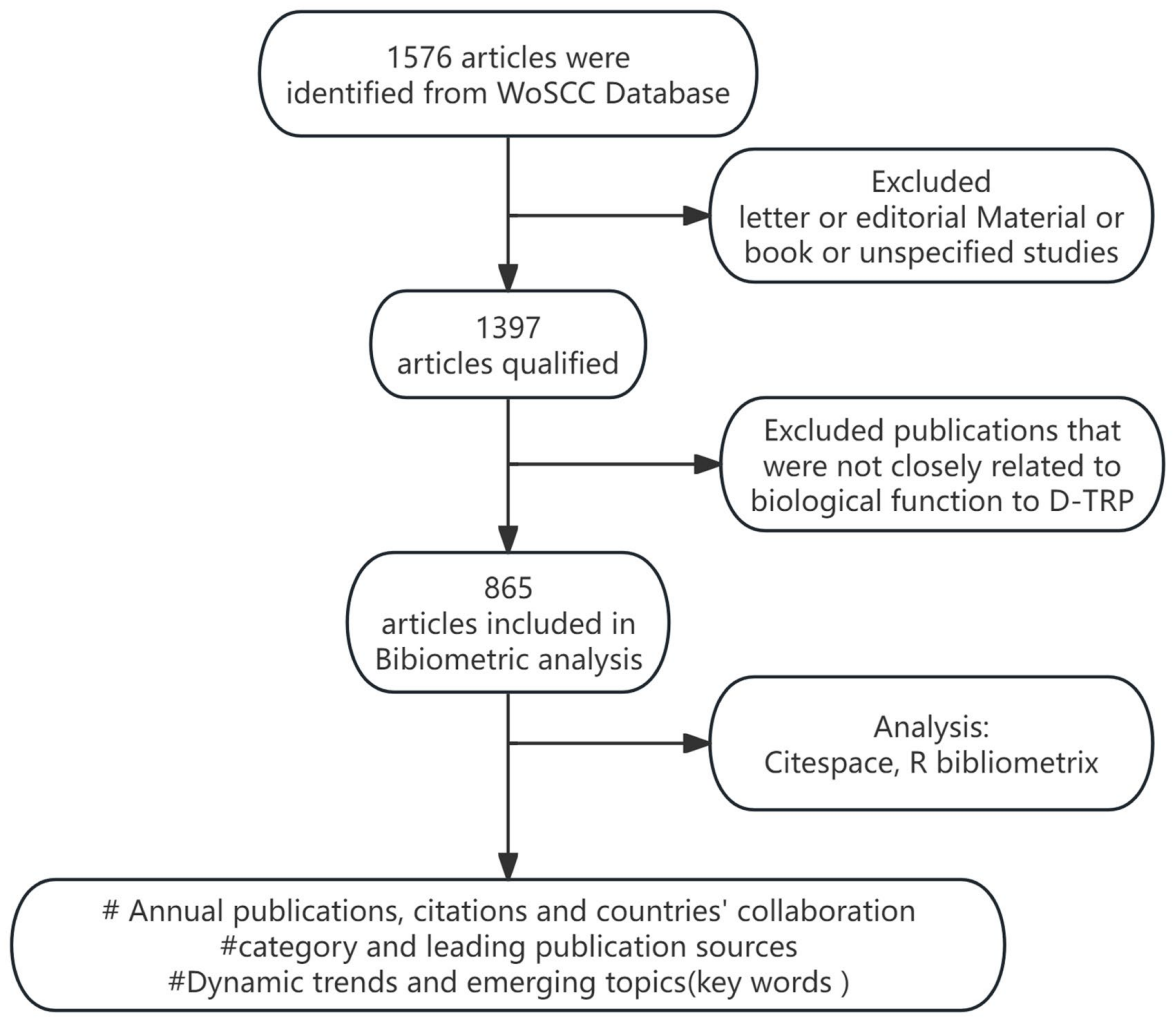
Workflow diagram. A total of 1,576 records were initially retrieved; further refinements eliminated the publications that were not closely related, resulting in a final collection of 865 publications. Data analysis was performed using “Bibliometrix” in R and CiteSpace.

From 1 January 1987 to 31 December 2023, 1,576 records were initially retrieved. Publications that were not closely related were eliminated, resulting in a final collection of 865 publications. Within this dataset, there were 2,209 keywords, 4,068 authors from 2094 institutions, and representatives from 302 regions. A workflow diagram is shown in Figure 1.

**Figure 2:**
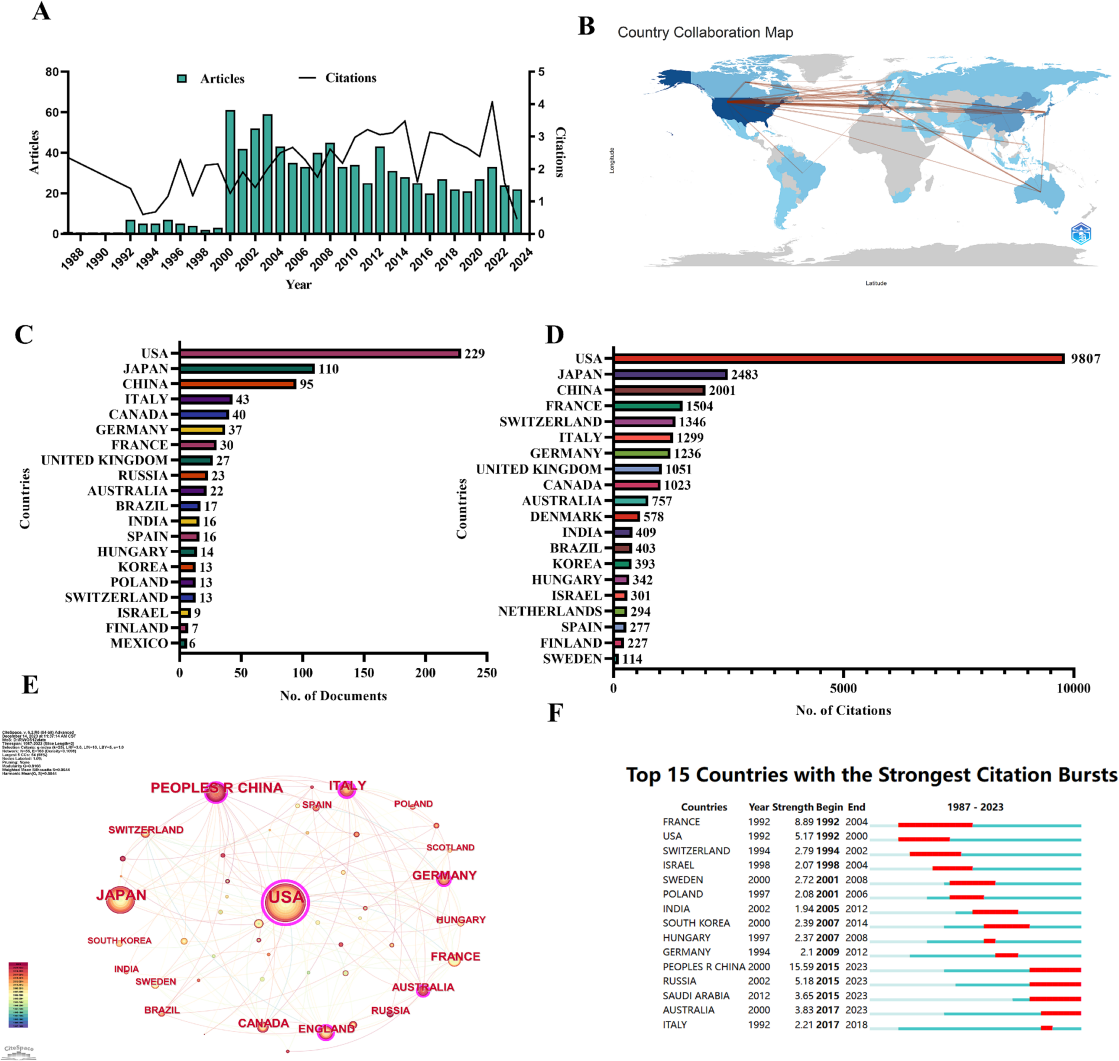
Number and citations of publications published on d-tryptophan. **(A)** Number and citations of publications published on d-tryptophan. **(B)** Collaboration WorldMap. **(C)** Number of publications in the top 20 most productive countries. **(D)** Number of citations in the top 20 most productive countries. **(E)** Country cooperation map from CiteSpace. **(F)** Countries with the strongest citation bursts.

The number of publications on d-tryptophan and the mean total number of citations per year over the past 37 years are shown in Figure 2A. As shown, the number of publications increased sharply in 2000, indicating that d-tryptophan research received increasing attention from researchers. The number of publications peaked in 2000, with 61 articles published, and the mean total citations per year peaked in 2014 at 3.16 with an average number of citations per document of 32.03.

The Collaboration WorldMap, as shown in Figure 2B, represents a map of international cooperation between countries and shows that the United States of America (United States) works closely with Japan and China. Among these countries, the USA had the most published papers (NP = 229, 26.5%), with 9,807 citations, followed by Japan (NP = 110, 12.7%), with 2,483 (Supplementary Table S1). Figure 2C shows the numbers of publications in the top 20 most productive countries, whereas Figure 2D shows the number of citations in the top 20 most productive countries. The United States, Japan, China, and Italy collaborated most (Figure 2E). In addition, the United States and France were the first countries to conduct studies of d-tryptophan. After 2000, the number of articles from Switzerland, Poland, and other countries increased. After 2015, citations from China, Russia, Saudi Arabia, and Australia increased (Figure 2F).

#### 2.5.2 Analysis of published journals

**Figure 3:**
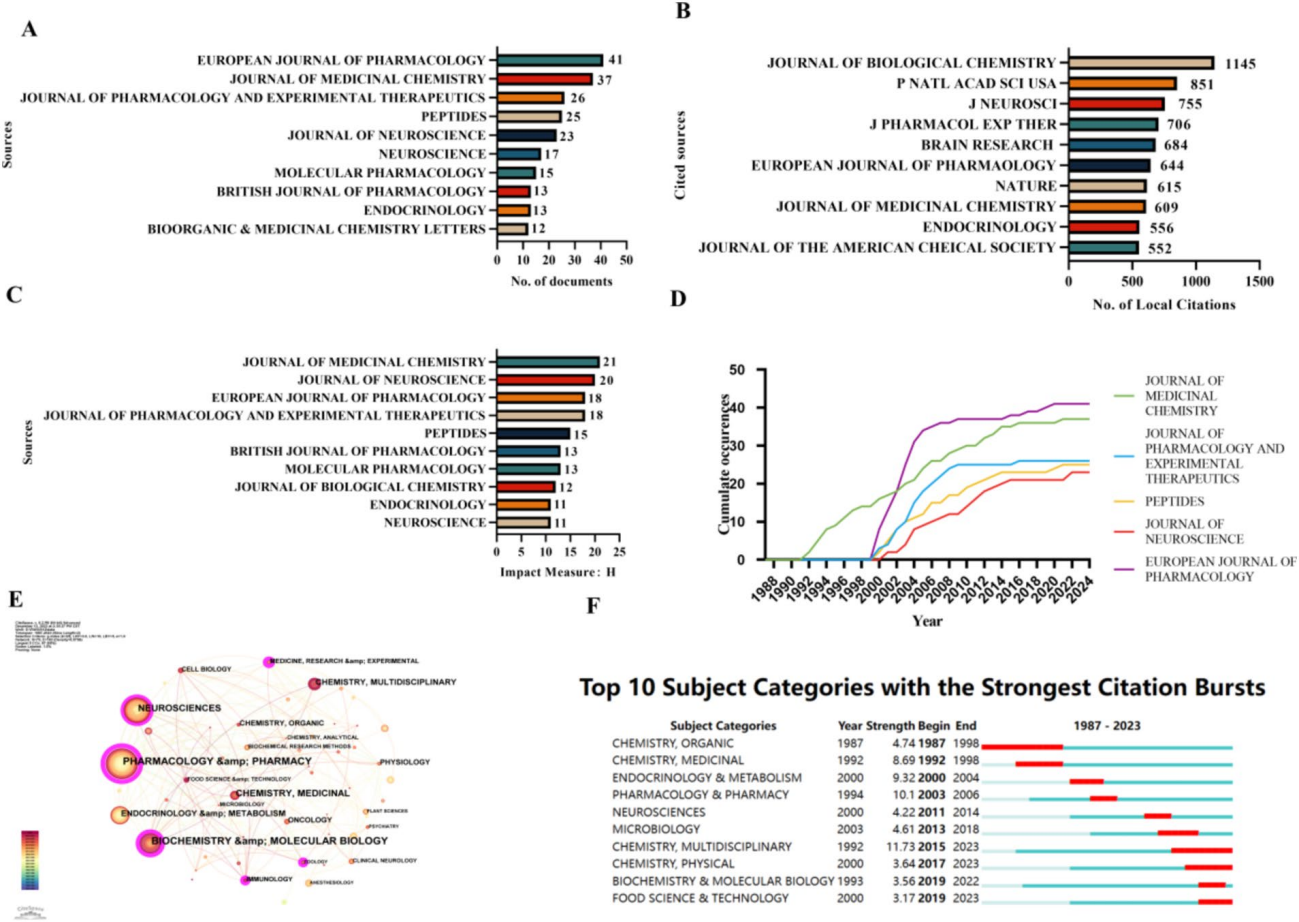
Analysis of published journals. **(A)** Top 10 most relevant sources. **(B)** Top 10 most locally cited sources. **(C)** Local impact of the top 10 sources. **(D)** Cumulative occurrences of the top five journals over time. **(E)** Co-occurrence of categories. **(F)** Subject categories exhibiting strong citation bursts.

The citation relationships among countries were analyzed using CiteSpace. The most relevant sources, most locally cited sources, and the local impact of the source on the top 10 are illustrated in Figure 3. The European Journal of Pharmacology led the discourse by publishing a substantial body of articles with 41 articles followed by the Journal of Medicinal Chemistry with 37 articles (Figure 3A). The Journal of Biological Chemistry was the most recurrently co-cited publication, having a frequency of 1,145, followed by Proceedings of the National Academy of Sciences of the United States of America (PNAS), having a frequency of 851 (Figure 3B). The Journal of Medical Chemistry had the highest impact index, H, of 21, followed by the Journal of Neuroscience, which had an H of 20 (Figure 3C). The cumulative occurrences of the top five journals over time are shown in figure 3D. Journal of Medicinal Chemistry started publishing articles on d-tryptophan in 1990; Journal of Pharmacology and Experimental Therapeutics, Peptides, Journal of Neuroscience, and European Journal of Pharmacology have published a large number of articles on d-tryptophan since 2000 (Figure 3D).

The fields of the published articles included medicinal chemistry, pharmacology, and neuromedicine (Figure 3E). Since 1987, organic and medicinal chemistry have become popular areas of research. Since 2015, research on d-tryptophan has been more multidisciplinary, focusing mainly on physiological functions and food science (Figure 3F).

#### 2.5.3 Keyword analysis

**Figure 4:**
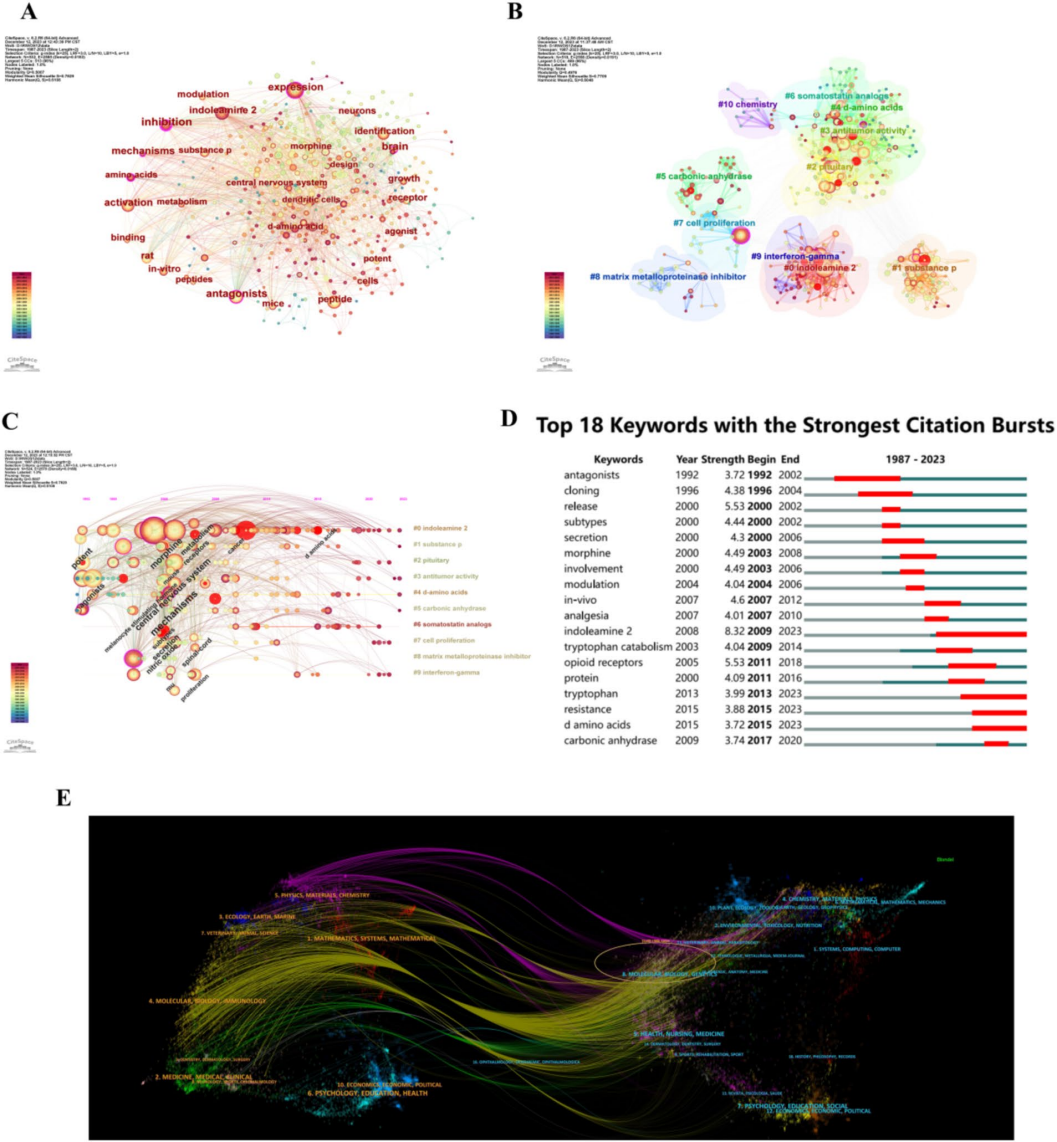
Analysis of keywords and dual map overlay of journals. **(A)** Co-occurrence of keywords. **(B)** Cluster analysis of keywords. **(C)** Timeline analysis of keywords. **(D)** Keywords exhibiting strong citation bursts. **(E)** Dual map overlay of journals.

The keywords of a paper are a highly concise summary of the research aims, subjects, and methods. As a result, a keyword-based analysis can reflect trends in study focus and research hot topics over a certain period. Using d-tryptophan/d-tryptophan/d-Trp/d-tryptophan/d-TRP/d-TRP as keywords, as shown in Figure 4, the most common keywords were “expression,” “indoleamine 2,” “amino acids,” “dendritic cells,” “d-amino acids,” “activation,” and “inhibition.” Keyword cluster analysis revealed that research focused on “indoleamine 2,” “substance p,” “antitumor activity,” “pituitary,” and “d-amino acids.” The analysis of timeline and keywords with the strongest citation bursts indicated that research on d-tryptophan started in 1992. Initially, the focus was on antagonists and subtypes, followed by the exploration of resistant d-amino acids and carbonic anhydrase. A dual-map overlay of journals publishing d-tryptophan research is shown in Figure 4D, revealing three main citation pathways: papers published in medicine/medical/clinical, molecular/biology/immunology, and molecular/biology/immunology journals were often cited in the papers published in environmental/toxicology/nutrition and health/nursing/medicine journals (Figure 4E).

## 3 d-Tryptophan: literature review

### 3.1 Characteristics of d-tryptophan

d-Tryptophan is a white solid with boiling and melting points of 447.91°C and 282–285°C, respectively. Its molecular formula and molecular weight are C_11_H_12_N_2_O_2_ and 204.22, respectively (Kepert et al., 2017). d-Tryptophan is soluble in water, and the resulting solutions have a pH of less than 7.0, thus acting as acids to neutralize bases. d-Tryptophan belongs to the class of organic compounds known as indolyl carboxylic acids and derivatives and is present in all living species. d-Tryptophan is found at the highest concentrations in cow milk and beer (Kolodkin-Gal et al., 2010). In eukaryotic systems, d-tryptophan may be primarily derived from microorganisms that synthesize different enantiomers and incorporate them into the cell wall (Kolodkin-Gal et al., 2010). Studies suggest that the small amounts of d-tryptophan detected in humans may originate from microorganisms in the digestive tract (Visser et al., 2011).

### 3.2 The metabolism of d-tryptophan

**Figure 5:**
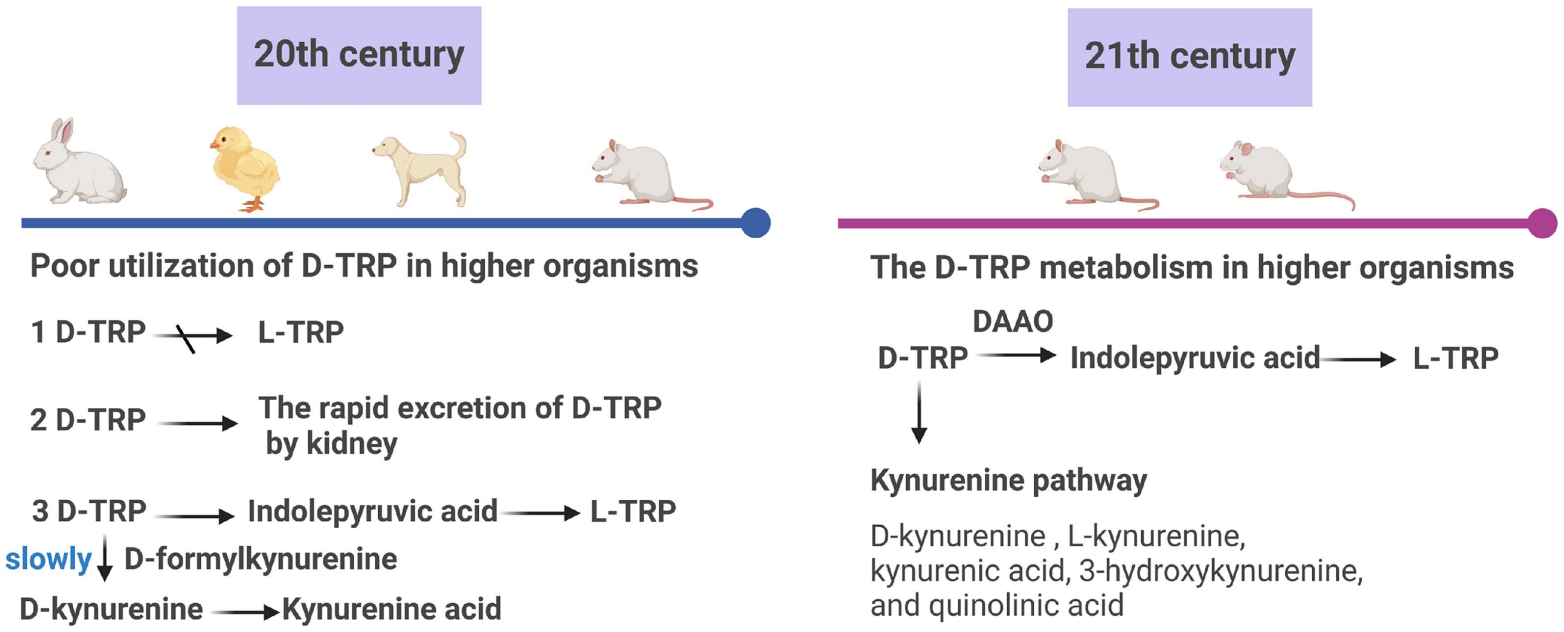
Research on the metabolism of d-tryptophan (d-TRP) in the 20th and 21st centuries. l-TRP: l-Tryptophan, DAAO: d-amino acid oxidase (created with biorender.com).

D-Tryptophan is a secondary metabolite, often serving as defense or signaling molecules. Studies have explored its metabolism, summarized in Figure 5. Early experiments showed limited utilization of D-tryptophan in higher organisms, with rapid clearance from plasma (Langner and Berg, 1955; Loh and Berg, 1971; Nesheim et al., 1974; Triebwasser et al., 1976). Loh and Berg found slow conversion of D-kynurenine (D-KYN) to kynurenic acid and potential formation of L-tryptophan from indole pyruvic acid in rabbits (Loh and Berg, 1971). Nesheim et al. observed rapid excretion of D-tryptophan in chick urine, partially attributed to kidney excretion (Nesheim et al., 1974). Studies in dogs by Budny et al. and Triebwasser et al. yielded differing conclusions; Budny found no significant conversion to L-tryptophan, while Triebwasser observed major urinary metabolites as unconverted D-tryptophan, D-kynurenine, and kynurenic acid, with inversion to L-tryptophan as a major product (Budny et al., 1976; Triebwasser et al., 1976). An enzyme system catalyzes conversion to D-kynurenine (Higuchi and Hayaishi, 1967; Tashiro et al., 1961). Ohara et al. found conversion to the L-isomer in rats after stomach intubation (Ohara et al., 1980).

In 2011, studies in rats showed that intraperitoneal administration of D-tryptophan resulted in inversion to L-tryptophan, increasing plasma concentration, while pre-treatment with an inhibitor of d-amino acid oxidase (DAAO) suppressed L-tryptophan levels (Iizuka et al., 2011). In 2016, systemic administration of D-tryptophan to mice revealed metabolism in both brain and peripheral systems, converting to kynurenine pathway metabolites, with DAAO involvement confirmed (Notarangelo et al., 2016). D-Kynurenine is a precursor to neuroactive compounds linked to neurological and psychiatric diseases (Wang et al., 2013).

### 3.3 Biological function

d-Amino acids are formed during food processing, originating from microbial sources and aqueous, soil, and other environments, and, thus, may become part of the human diet (Friedman, 1999). As a non-protein amino acid, d-tryptophan has unique physiological properties and can be used as a non-nutritional sweetener, food additive, immunomodulatory probiotic substance, and for developing drugs.

#### 3.3.1 Nutritional effects

D-Tryptophan, indeed, possesses numerous nutritional benefits. Many probiotics and prebiotics play a role in the body, and metabolomics studies have found that D-tryptophan levels were elevated. The rice bran dietary fiber which has a variety of potential health benefits, especially its probiotic effects on gut health, have been shown to significantly alter the meta bolic profile of the gut microbiota, including notable downregulation of l-pyroglutamic acid and sulfolithocholic acid and upregulation of D-tryptophan and 4-hydroxybenzaldehyde (Cheng et al., 2024). Untargeted metabolomics analyzed the influence of *Lactobacillus plantarum* CCFM8610 on germ-free mice and found that five metabolites, L-methionine, D-tryptophan, indoleacrylic acid, DL-acetylcarnitine, and L-norleucine, were identified as key metabolites in the serum (Huang et al., 2023). In a series of four experiments spanning 28 days each, weanling pigs were used to assess the nutritional substitution of d-tryptophan for l-tryptophan (Arentson and Zimmerman, 1985). The addition of both d- and l-tryptophan to diets lacking in tryptophan notably enhanced the pigs’ feed consumption, growth rate, and weight gain while reducing plasma urea nitrogen levels. Further, d-tryptophan supplementation proved to be around 70% as effective as that of l-tryptophan in boosting the average daily feed intake and the ratio of weight gain to feed in pigs consuming diets low in tryptophan derived from natural ingredients (Arentson and Zimmerman, 1985).

Contrastingly, some studies have indicated that d-tryptophan has limited nutritional value. For instance, the inclusion of d-tryptophan into a lysine-supplemented wheat gluten diet, which otherwise maintains a nutritional status in infants like that provided by evaporated milk formula, could lead to undesirable nutritional outcomes (Albanese and Snyderman, 1949). Additionally, the Na^+^-independent absorption of l-tryptophan into the jejunal vesicles of 21-day-old chicks was found to be reduced in the presence of d-tryptophan compared to when 2-aminobicyclo-[2,2,1]-heptane-2-carboxylic acid was present (Iji et al., 2001). Diets with high levels of d-tryptophan have also been shown to impede mouse growth (Friedman, 1999; Friedman, 2010). The negative effects of d-tryptophan, as well as its viability as an indirect measure for forecasting detrimental effects in rats, have also been explored (Shibata et al., 2014). In one study, male rats were given various test diets containing 20% casein with incremental concentrations of d-tryptophan (0–0.5%) for 21 days. Urine samples collected over 24 h on the experiment’s final day revealed that diets with 0.3% d-tryptophan led to observable adverse effects on food intake and body weight (Shibata et al., 2014).

In addition, when d-tryptophan, which is a sweet-tasting amino acid, was fed to rats, the release of glucose-dependent insulinotropic polypeptide and glucagon-like peptide-1 did not occur. Crucially, these peptides are typically secreted during meals by endocrine cells in the gut mucosa and act to stimulate insulin release from pancreatic *β*-cells in response to glucose (Fujita et al., 2009). Thus, the increase in incretin secretion from the gut triggered by sugar was not observed on administration of d-tryptophan *in vivo* (Fujita et al., 2009). Furthermore, variations in the consumption of certain sweet-tasting amino acids (d-phenylalanine, d-tryptophan, and l-proline) among mouse strains have been linked to polymorphisms in the taste receptor type-1 member 3 gene (Tas1r3), suggesting differences in peripheral taste sensitivity (Bachmanov et al., 2016).

#### 3.3.2 Use of d-tryptophan as a preservative and for controlling bacterial growth in foods

d-Tryptophan is recognized for its ability to suppress the growth of certain bacteria, making it a valuable preservative for food (Chen et al., 2022; Elafify et al., 2022a; Elafify et al., 2022b; Koseki et al., 2015).The rapid spoilage of fresh seafood is largely due to microbial activity. Given the reluctance of consumers to accept common antimicrobials and synthetic food additives, which can compromise food quality and health, novel preservation methods are required. These methods could be standalone or combined with traditional techniques such as chilling, salting, and freezing. d-Tryptophan has shown promise as a novel preservative, particularly in salmon fillets (Chen et al., 2022). When used alongside salt, d-tryptophan effectively inhibits the growth of *Shewanella baltica* and *Pseudomonas fluorescens* by reducing their respiratory ability; high levels of NaCl (>3.5%) with d-tryptophan (>15 mM) are required for achieving optimal growth inhibition (Chen et al., 2022).

In the dairy sector, d-tryptophan is emerging as a favorable alternative (Elafify et al., 2022b). Its addition (40 mM) to soft cheese and ice cream contaminated with *Escherichia coli O26:H11* resulted in a significant reduction in bacterial proliferation, especially when combined with other stressors found in food (Elafify et al., 2022b). Similarly, treating soft cheese contaminated with *Salmonella* using a blend of pomegranate peel extract, ascorbic acid, and d-tryptophan led to a dose-dependent decrease in bacterial growth (Elafify et al., 2022a).

The impact of d-tryptophan varies across different bacterial species, highlighting the differences between gram-positive and gram-negative bacteria (Koseki et al., 2015). At a concentration of approximately 40 mM and in a peptone–yeast–glucose broth with 0–4% salt (w/v), d-tryptophan inhibited the growth of *Listeria monocytogenes*, *Salmonella enterica*, and *Escherichia coli O157:H7* at 25°C (Koseki et al., 2015). Concentrations of 30–40 mM d-tryptophan completely inhibited the growth of *Escherichia coli O157:H7* and *Salmonella* in environments with >3% salt, although the growth of *Listeria monocytogenes* was not entirely suppressed under similar conditions (Koseki et al., 2015). d-Tryptophan notably inhibited the growth of gram-negative bacteria. The efficacy of d-tryptophan as an antibacterial agent against *Escherichia coli* is modulated by the concentration of NaCl and the temperature (Kan et al., 2018). Under osmotic stress, the influence of d-tryptophan on the growth of *Listeria monocytogenes* in milk and cream is contingent on the level of temperature stress, whether cold and/or heat (Elafify et al., 2020). Employing exogenous d-tryptophan in marine settings has proven to be an effective method for controlling *Vibrio* in live oysters at room temperature and for delaying bacterial spoilage, thereby extending the shelf life of shucked oysters stored under refrigeration (Chen et al., 2018).These findings indicate the potential of d-tryptophan as an innovative food preservative to manage bacterial growth in various food products (Koseki et al., 2015). In most studies, the use of 40 mM d-tryptophan, along with other stress conditions, had excellent antimicrobial effects on food-borne pathogens (Moghimani et al., 2024).

There is a lack of direct information on the synergistic mechanism of D-tryptophan with traditional preservatives such as salt and refrigeration, as well as its impact on consumer acceptance. Future research is needed to explore the specific interactions and benefits of D-tryptophan in combination with traditional preservatives.

#### 3.3.3 Inhibition of bacterial biofilms

The capacity of bacterial pathogens to create biofilms is a significant aspect of their virulence, influencing their disease progression and spread (Elgamoudi et al., 2020). Biofilms are vital for microbial endurance in adverse conditions and serve as repositories for microbial contamination and antibiotic resistance (Elgamoudi et al., 2020).

The formation of biofilms by *Pseudomonas* and *Staphylococcus*, which leads to biofouling in natural environments, can be hindered by d-tryptophan. Therefore, this compound is being considered for biofouling control applications (Ghosh et al., 2019). Experiments have shown that d-tryptophan significantly reduces the attachment and biofilm formation rates of these bacteria on polystyrene 96-well microtiter plates compared to l-tryptophan or a mix of d−/l-tryptophan, emphasizing its potential utility in developing surface coating technologies (Ghosh et al., 2019). *Bifidobacterium longum* (BL21) has been noted to decrease the *Firmicutes/Bacteroidetes* ratio, affect liver remodeling in glycerophospholipids, and reduce d-tryptophan levels (Wu et al., 2020). It also offers protection against *Roseburia* biofilms by inhibiting d- tryptophan production (Kolodkin-Gal et al., 2010).

*Bacillus subtilis* produces a mix of d-leucine, d-methionine, d-tyrosine, and d-tryptophan that not only prevents biofilm formation but also disassembles existing biofilms (Kolodkin-Gal et al., 2010). *Enterococcus faecalis*, commonly found in post-treatment diseases and a key player in persistent infections after root canal procedures, can be combated by incorporating d-amino acids into endodontic treatments to reduce its biofilm formation (Zilm et al., 2017). An equimolar mix of d-methionine, d-tyrosine, d-leucine, and d-tryptophan at 50 ppm significantly improved the efficacy of a 50 ppm tetrakis (hydroxymethyl) phosphonium sulfate biocide treatment against stubborn biofilm consortia from oil-field operations, which include sulfate-reducing, nitrate-reducing, and fermentative bacteria (Li et al., 2016). This mixture was found to inhibit biofilm formation by incorporating these d-amino acids into the bacterial cell wall (Leiman et al., 2013). The biofilm-dispersal mechanism may involve the effect of d-amino acids on the formation of amyloid-like fibrils within the biofilm matrix. Matrix-associated amyloid fibrils have been previously reported to form a part of *Campylobacter jejuni* biofilms (Turonova et al., 2016); similar d-amino acid–induced disassembly of matrix-associated amyloid fibers has also been reported for *Bacillus subtilis* biofilms (Cava et al., 2011). d-Amino acid treatment cause the release of amyloid fibers that linked cells in the biofilm together; additionally, the mutants that are able to form biofilms in the presence of d-amino acids contain alterations in a protein (YqxM) required for the formation and anchoring of the fibers to the cell (Cava et al., 2011; Kolodkin-Gal et al., 2010). The presence of l-alanine and d-tryptophan reduces the transcript levels of peptidoglycan biosynthesis enzyme alanine racemase in *Campylobacter jejuni,* leading to the inhibition of growth and biofilm formation (Elgamoudi et al., 2020).

#### 3.3.4 Immunomodulatory probiotic properties

**Figure 6:**
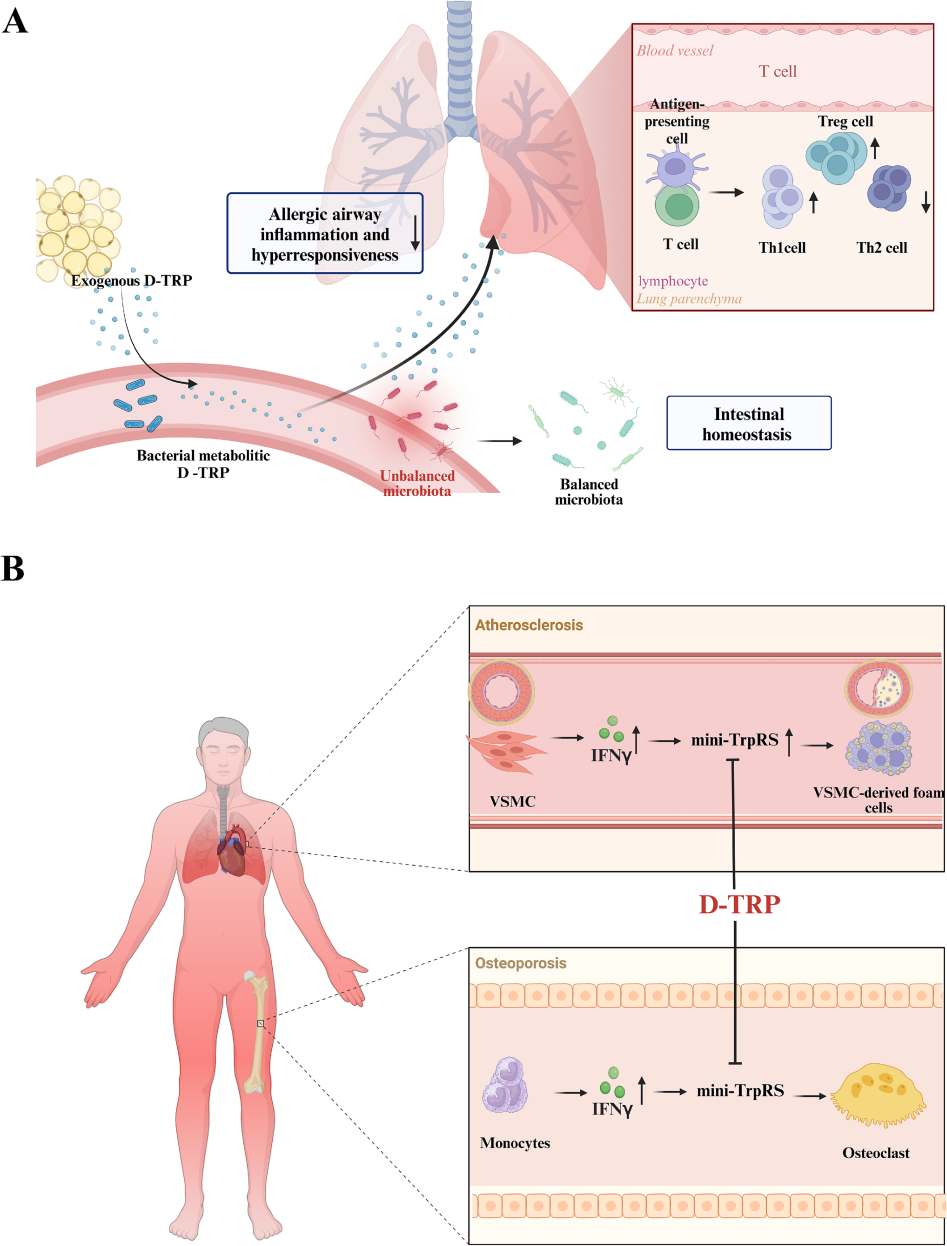
Biological function of d-tryptophan (d-TRP). **(A)** d-TRP as an immunomodulatory probiotic substance. **(B)** d-TRP reduces multinucleation by blocking the IFN-*γ*-induced mini-TrpRS and plays a role in the treatment of atherosclerosis and osteoporosis (created with biorender.com).

d-Amino acids, synthesized by gut bacteria, serve as powerful bactericidal agents with diverse roles in both mammals and microbes (Sasabe and Suzuki, 2018). d-Phenylalanine and d-tryptophan are involved in directing neutrophil movement via a G-protein-coupled receptor, whereas d-serine plays a bacteriostatic role in the urinary system. d-phenylalanine and d-leucine can suppress innate immunity through the sweet taste receptor in the upper respiratory tract, and d-tryptophan is known to modulate immune tolerance in the lower respiratory tract (Sasabe and Suzuki, 2018). These amino acids, including d-kynurenine, a metabolite of d-tryptophan, reduce adenylate cyclase activity in cells expressing GPR109B cDNA by activating PTX-sensitive G proteins and serve as attractants for blood cells (Irukayama-Tomobe et al., 2009).

d-Tryptophan, which, as mentioned, is a product of microbial synthesis, is recognized for its immunomodulatory effects. It has been shown to maintain intestinal balance and is considered a prebiotic with potential benefits for allergic conditions (Figure 6A).

Administering d-tryptophan to mice before inducing asthma increases regulatory T cells in the lungs and gut, reduces lung Th2 responses, and mitigates allergic airway inflammation and hyperresponsiveness (Kepert et al., 2017). Further, research has indicated that d-tryptophan can influence the balance between T helper cell types 1 and 2 in an asthma model induced by ovalbumin, with radical S-adenosyl methionine domain-containing 2 identified as possible target for d-tryptophan (Wang and Shang, 2024). d-Tryptophan significantly promotes interleukin (IL)-10 production and reduces the secretion of lipopolysaccharide-induced IL-12 and IL-5 in human Hodgkin’s disease cell line cultures, thereby preventing the onset of allergic airway conditions (Kepert et al., 2017).

Characteristic of certain probiotic strains such as *Lactobacillus rhamnosus GG, Lactobacilli casei W56, and Bacillus subtilis* (Zhang J. et al., 2021), d-tryptophan hinders the growth of harmful gut and colitis-inducing pathogens, offering protection against fatal *Citrobacter rodentium* infections by diminishing the pathogen load (Seki et al., 2022). It also prevents the development of experimental colitis by eliminating specific intestinal microbes, increases the intracellular level of indole acrylic acid (IA), a crucial determinant of microbial susceptibility to d-tryptophan, and acts as a modulator of the gut environment, thereby regulating intestinal homeostasis (Seki et al., 2022). Elevated levels of d-tryptophan have been observed in B6DAO mice lacking DAAO, which is responsible for its breakdown (Ishii et al., 2022). The aryl hydrocarbon receptor (AhR) plays a key role in mediating the body’s response to external compounds, and DAAO aids in producing AhR activators by converting d-tryptophan through enzymatic reactions (Chowdhury et al., 2009). This process results in the formation of potent AhR stimulators such as 1,3-di(1H-indol-3-yl)propan-2-one and 1-(1H-indol-3-yl)-3-(3H-indol-3-ylidene) propan-2-one, which can activate the AhR at very low concentrations (Chowdhury et al., 2009). Additionally, the production of I3P from amino acid precursors can modulate endogenous AhR activity through various enzymatic pathways (Nguyen et al., 2009).

#### 3.3.5 Applications of d-tryptophan in atherosclerosis and osteoporosis

d-Tryptophan is pivotal in managing atherosclerosis and osteoporosis. The IFN-*γ*/miniTrpRS (mini-tryptophanyl-tRNA synthetase) axis is implicated in both atherosclerosis (Biros et al., 2022b) and osteoporosis (Biros et al., 2022a). d-tryptophan, a cognate amino acid and decoy substrate for mini-TrpRS, reduces multinucleation by blocking the IFN-γ-induced mini-TrpRS (Biros et al., 2021; Figure 6B).

In atherosclerosis, vascular smooth muscle cells (VSMCs) exhibit plasticity, transitioning from a contractile to a synthetic phenotype (Elmarasi et al., 2024). VSMC-derived foam cells, which constitute a significant portion of all foam cells, are dependent on this phenotypic switch, a critical step in disease progression (Biros et al., 2022b). d-tryptophan, also known as indoximod, is a promising candidate for controlling atherosclerosis due to its patient safety profile and its ability to inhibit mini-TrpRS activation by IFN-γ (Biros et al., 2022b). Circulating IFN-γ levels were significantly increased in patients with osteoporosis; additionally, upregulation of IFN-γ is associated with severe osteoporotic phenotypes such as osteoporotic fractures (Pietschmann et al., 2001). In osteoporosis, direct blockade of mini-TrpRS in the presence of IFN-γ impacts the multinucleation of monocytes to limit osteoclast formation (Biros et al., 2021). In osteoporosis, d-tryptophan does not engage in the KYN/AhR/RANKL pathway, as indoleamine 2,3-dioxygenase 1 (IDO1) cannot process the d-form of tryptophan; additionally, its effects on the IDO1/KYN/AhR pathway are secondary to its primary action on the IFN-γ/mini-TrpRS axis during the early stages of osteoclastogenesis (Kim et al., 2012). The role of indoximod in preventing osteoclast formation underscores its potential as a therapeutic agent for osteoporosis (Biros et al., 2022a).

#### 3.3.6 Applications of d-tryptophan as a drug

D-Tryptophan is also under investigation as a drug, specifically its prodrug form NLG8189, known as 1-methyl-d-tryptophan (1-MT) (Kumar et al., 2020; Prendergast et al., 2017) developed by Lumos Pharma. This compound is linked to the treatment of various conditions, including infections (Potula et al., 2005), tuberculosis (Singh et al., 2023), and cancer (Johnson et al., 2024; Li et al., 2022; Matheus et al., 2020).

IDO, an enzyme that regulates tryptophan metabolism and the immune system, is crucial for maintaining immune tolerance and modulating immune responses (Stone and Williams, 2023a; Stone and Williams, 2023b). Its activity is associated with a range of conditions, including cancer (Qian et al., 2023), autoimmune diseases (Zhao et al., 2022), and infectious diseases (Xie et al., 2023). 1-MT, as an IDO inhibitor, has complex inhibitory effects on these processes (Fox et al., 2018). In the context of HIV-1 encephalitis, 1-MT influences the production of cytotoxic T lymphocytes and aids in the clearance of virus-infected macrophages, as demonstrated in animal models (Potula et al., 2005). Furthermore, nanoformulated indoximod has been shown to improve ulcerative colitis by enhancing mitochondrial function and facilitating mucosal repair (Wu et al., 2023).

IDO expression is markedly increased Indian origin rhesus macaque tuberculosis granulomas, particularly in cells responsive to interferon and myeloid-derived suppressor cells (Singh et al., 2023). The compound 1-MT can inhibit IDO, leading to a restructured granuloma with a higher recruitment of T cells to the core of the lesion, suggesting its potential as a supplementary treatment alongside anti-TB chemotherapy in clinical settings (Singh et al., 2023).

Matheus et al. (2020) indicated that 1-MT can activate the AhR, which is linked to the progression of bladder cancer. The GA-1MT molecule, a combination of 1-MT and gallic acid, has been shown to suppress melanoma growth effectively by inhibiting tyrosinase expression and modulating T cell populations within tumors, outperforming the individual effects of gallic acid and 1-MT (Liu H. et al., 2022). A novel nanoparticle delivery system that combines photothermal therapy and immunotherapy, incorporating 1-MT and Toll-like receptor agonists into polydopamine nanoparticles, has demonstrated efficacy in halting the growth of mouse breast carcinoma cells, promoting apoptosis, and enhancing immune responses (Li et al., 2022). The use of oxaliplatin with 1-MT has significantly reduced tumor growth, extended the lifespan of mice with tumors, and facilitated T cell infiltration into tumor tissues (Zhang Y. et al., 2021). A phase I trial by Johnson et al. found that the oral IDO-pathway inhibitor indoximod is well-tolerated in children with recurrent brain tumors or newly diagnosed diffuse intrinsic pontine glioma and can be safely used with chemotherapy and radiation (Johnson et al., 2024). Additionally, dibenzocyclooctyne+programmed death-1 nanovesicles carrying 1-MT for targeted cancer immunotherapy have shown to significantly reduce tumor growth and improve survival in mice (Xu et al., 2022). The majority of the aforementioned experiments rely on animal studies that involve limited sample sizes, necessitating further clinical research to definitively ascertain the role of D-tryptophan.

#### 3.3.7 d-Tryptophan’s role in sexual induction and reproductive development in planarians

In the realm of developmental biology, d-tryptophan is crucial for the sexual induction of the planarian *Dugesia ryukyuensis*, a model organism for studying postembryonic germ cell development (Kobayashi et al., 2017). The amino acid transporter gene Dr-SLC38A9, highly expressed in sexual worms and various reproductive tissues, is essential for the development of reproductive organs, with its expression being upregulated by d-tryptophan (Maezawa et al., 2021). This highlights the significant role of d-tryptophan in both immunomodulation and the regulation of reproductive development in certain organisms.

This study provides a bibliometric analysis of D-tryptophan research over the past decades. It illustrates the transition from the study of basic metabolism to the exploration of its potential applications in the fields of food science and medicine. Nevertheless, there are a few areas in which improvements could be made. This bibliometric analysis relied solely on the WoSCC database and included only English-language publications, which potentially excluding relevant research in other languages. The metrics used (such as citation counts) may be subject to discipline and journal influence. However, WoSCC with its high-quality interdisciplinary coverage, robust citation tracking, and de-duplication functions, significantly enhances the efficiency and accuracy of bibliometric studies, thereby meeting the research needs of the medical field (Gusenbauer, 2024). Future bibliometric studies could incorporate additional databases such as Scopus or PubMed to minimize potential language and regional biases.

Despite its promising potential, the use of D-tryptophan in treating cancer, osteoporosis, and atherosclerosis remains largely experimental. Further clinical trials are crucial to validate its safety and efficacy across various conditions. D-Tryptophan has demonstrated significant roles in food preservation, inhibiting bacterial biofilms, immunomodulation, managing atherosclerosis and osteoporosis, and as a precursor for 1-MT. Future research should focus on examining the stability and effectiveness of D-tryptophan in complex food systems. Multi-center randomized controlled trials should be designed to verify its safety and efficacy in treating cancer and metabolic diseases. Moreover, the molecular mechanism of D-tryptophan in the AhR and its role in regulating intestinal flora remain understudied areas that deserve further.

## 4 Conclusion

In this study, we explored the research trends in d-tryptophan from 1987 to 2023 through a bibliometric analysis. d-Tryptophan has unique physiological properties and value in the fields of biochemistry, pharmacology, microbiology, and other areas and is a key research subject in the food, feed, and pharmaceutical industries. In recent years, research on d-tryptophan has mainly focused on multidisciplinary research, including biochemistry, pharmacology, microbiology, food, and neurophysiological functions. In addition, d-tryptophan is considered to have immunomodulatory, anti-tumor, anti-viral, and other effects and is a hot topic for future research.

## Data Availability

The original contributions presented in the study are included in the article/Supplementary material, further inquiries can be directed to the corresponding author/s.
