# Supplementary material for: Biological function of d-tryptophan: a bibliometric analysis and review
Source: Front Microbiol. 2025 Jan 13;15:1455540. doi: 10.3389/fmicb.2024.1455540 (PMC11770058; doi:10.3389/fmicb.2024.1455540)
Supplement: Supplementary file 1 [file Table_1.docx]

Supplementary Material

**Supplement table 1. Top 20 most prolific countries in the field of d-tryptophan research.**

| Country | Articles | SCP | MCP | Freq | MCP_Ratio | Country | TC | AAC |
| --- | --- | --- | --- | --- | --- | --- | --- | --- |
| USA | 229 | 177 | 52 | 0.265 | 0.227 | USA | 9807 | 42.80 |
| JAPAN | 110 | 97 | 13 | 0.127 | 0.118 | JAPAN | 2483 | 22.60 |
| CHINA | 95 | 72 | 23 | 0.11 | 0.242 | CHINA | 2001 | 21.10 |
| ITALY | 43 | 22 | 21 | 0.05 | 0.488 | FRANCE | 1504 | 50.10 |
| CANADA | 40 | 24 | 16 | 0.046 | 0.4 | SWITZERLAND | 1346 | 103.50 |
| GERMANY | 37 | 24 | 13 | 0.043 | 0.351 | ITALY | 1299 | 30.20 |
| FRANCE | 30 | 20 | 10 | 0.035 | 0.333 | GERMANY | 1236 | 33.40 |
| UNITED KINGDOM | 27 | 14 | 13 | 0.031 | 0.481 | UNITED KINGDOM | 1051 | 38.90 |
| RUSSIA | 23 | 22 | 1 | 0.027 | 0.043 | CANADA | 1023 | 25.60 |
| AUSTRALIA | 22 | 16 | 6 | 0.025 | 0.273 | AUSTRALIA | 757 | 34.40 |
| BRAZIL | 17 | 15 | 2 | 0.02 | 0.118 | DENMARK | 578 | 115.60 |
| INDIA | 16 | 14 | 2 | 0.018 | 0.125 | INDIA | 409 | 25.60 |
| SPAIN | 16 | 14 | 2 | 0.018 | 0.125 | BRAZIL | 403 | 23.70 |
| HUNGARY | 14 | 8 | 6 | 0.016 | 0.429 | KOREA | 393 | 30.20 |
| KOREA | 13 | 11 | 2 | 0.015 | 0.154 | HUNGARY | 342 | 24.40 |
| POLAND | 13 | 11 | 2 | 0.015 | 0.154 | ISRAEL | 301 | 33.40 |
| SWITZERLAND | 13 | 7 | 6 | 0.015 | 0.462 | NETHERLANDS | 294 | 73.50 |
| ISRAEL | 9 | 8 | 1 | 0.01 | 0.111 | SPAIN | 277 | 17.30 |
| FINLAND | 7 | 4 | 3 | 0.008 | 0.429 | FINLAND | 227 | 32.40 |
| MEXICO | 6 | 5 | 1 | 0.007 | 0.167 | SWEDEN | 114 | 19.00 |

The top 20 most prolific countries in the field of d-tryptophan research, as well as their total number of publications (NP), number of papers co-authored with authors from other countries (MCP), number of papers co-authored by authors of the same nationality (SCP), ratio of international cooperation (MCP_Ratio), total citations, and average citations. NP: total number of publications, MCP: number of co-authored papers with authors from other countries, SCP: number of papers co-authored by authors of the same nationality, MCP_Ratio: ratio of international cooperation, TC: Total Citations, AAC: Average article citations.

**Supplement table 2. Articles and citations of the journals.**

| Sources | Articles | Sources | Citations | Element | H index | TC | PY_start |
| --- | --- | --- | --- | --- | --- | --- | --- |
| EUROPEAN JOURNAL OF PHARMACOLOGY | 41 | JOURNAL OF BIOLOGICAL CHEMISTRY | 1145 | JOURNAL OF MEDICINAL CHEMISTRY | 21 | 1103 | 1992 |
| JOURNAL OF MEDICINAL CHEMISTRY | 37 | PROCEEDINGS OF THE NATIONAL ACADEMY OF SCIENCES OF THE UNITED STATES OF AMERICA | 851 | JOURNAL OF NEUROSCIENCE | 20 | 1450 | 2001 |
| JOURNAL OF PHARMACOLOGY AND EXPERIMENTAL THERAPEUTICS | 26 | JOURNAL OF NEUROSCIENCE | 755 | EUROPEAN JOURNAL OF PHARMACOLOGY | 18 | 805 | 2000 |
| PEPTIDES | 25 | JOURNAL OF PHARMACOLOGY AND EXPERIMENTAL THERAPEUTICS | 706 | JOURNAL OF PHARMACOLOGY AND EXPERIMENTAL THERAPEUTICS | 18 | 910 | 2000 |
| JOURNAL OF NEUROSCIENCE | 23 | BRAIN RESEARCH | 684 | PEPTIDES | 15 | 662 | 2000 |
| NEUROSCIENCE | 17 | EUROPEAN JOURNAL OF PHARMACOLOGY | 644 | BRITISH JOURNAL OF PHARMACOLOGY | 13 | 333 | 2000 |
| MOLECULAR PHARMACOLOGY | 15 | NATURE | 615 | MOLECULAR PHARMACOLOGY | 13 | 682 | 2000 |
| BRITISH JOURNAL OF PHARMACOLOGY | 13 | JOURNAL OF MEDICINAL CHEMISTRY | 609 | JOURNAL OF BIOLOGICAL CHEMISTRY | 12 | 1049 | 2000 |
| ENDOCRINOLOGY | 13 | ENDOCRINOLOGY | 556 | ENDOCRINOLOGY | 11 | 438 | 2000 |
| BIOORGANIC & MEDICINAL CHEMISTRY LETTERS | 12 | JOURNAL OF THE AMERICAN CHEICAL SOCIETY | 552 | NEUROSCIENCE | 11 | 462 | 2002 |

TC: total citations, PY_start：start publication years.
